# Patients’ Experiences of Helpfulness in Guided Internet-Based Treatment for Depression: Qualitative Study of Integrated Therapeutic Dimensions

**DOI:** 10.2196/jmir.2531

**Published:** 2013-06-20

**Authors:** Kjersti R Lillevoll, Maja Wilhelmsen, Nils Kolstrup, Ragnhild Sørensen Høifødt, Knut Waterloo, Martin Eisemann, Mette Bech Risør

**Affiliations:** ^1^Department of PsychologyFaculty of Health SciencesTromsøNorway; ^2^Department of Community Medicine, General Practice Research UnitFaculty of Health SciencesUniversity of TromsøTromsøNorway; ^3^Department of PsychologyFaculty of Health SciencesUniversity of TromsøTromsøNorway

**Keywords:** Internet-based cognitive behavioral therapy, ICBT, guided self-help, depression, qualitative

## Abstract

**Background:**

Quantitative research on Internet-based cognitive behavioral therapy (ICBT) has collected substantial evidence for the effectiveness of this treatment approach on health outcomes. Less is known about how patients find ICBT to be generally meaningful and helpful for treating depression.

**Objective:**

To explore patients’ experiences of being in ICBT treatment with a focus on the treatment dimensions that they considered helpful.

**Methods:**

Choosing a phenomenological-hermeneutical approach, 14 patients were interviewed with semistructured qualitative interviews to elicit their understanding of using ICBT. The patients took part in a clinical trial using ICBT with MoodGYM, which also featured brief consultations with a clinical psychologist. The interviews were transcribed and analyzed according to the chosen methodology and organized into significant themes.

**Results:**

The phenomenological-hermeneutical analysis identified 5 themes relating overall to the meaning of this mode of treatment in terms of helpfulness. Two related to treatment in general: (1) taking action to address one’s problems and (2) the value of talking to a professional. The next two themes specifically addressed guided self-help using the MoodGYM program: (3) acquiring relevant knowledge, and (4) restructuring the new knowledge acquired through ICBT. A fifth theme concerned (5) actual changes in patients’ perceptions and interactions, related to either the self-help material or the face-to-face consultations with the therapist.

**Conclusions:**

Three important dimensions were made explicit: the active engagement of the patient, the guidance of the therapist, and the content of the treatment program. The findings pointed to (1) the role of MoodGYM as a source of new knowledge providing patients with a structured approach to work with their depression, (2) the patient’s role as the primary agent of change through adapting relevant knowledge from MoodGYM to their situation, and (3) the dialogue with the therapist as a trusting relationship in which to share thoughts and feelings, receive feedback and advice, and to assist the patient in making use of the MoodGYM content.

## Introduction

Research has yielded promising results on the effects of Internet-based cognitive behavioral therapy (ICBT) on a range of mental health problems, including depression [1-3]. This form of therapy has the advantage of increased availability and at the same time puts less strain on therapeutic resources [4]. ICBT can be unguided, meaning that the patient works alone with the self-help material; or it can be guided, meaning that the patient enjoys some support and guidance from a therapist. The research on ICBT and other computerized treatments to date indicates that guided self-help and traditional face-to-face-therapy may offer roughly the same success rates for health outcomes [5], but it points to the importance of providing users with support during self-help programs [2,3,6]. Still, the role of support is not well understood in terms of the amount necessary or what it should offer [7,8].

The mechanisms through which ICBT is effective in reducing depression remain unclear. Both specific and common factors of treatment may serve as active ingredients [9]. Cognitive behavioral therapy (CBT) aims to alter the maladaptive structures and processes fundamental to depression [10], by making use of both cognitive and behavioral strategies. A key assumption in CBT is that the depressive patient can use CBT to modify or deactivate his/her depressogenic schema or develop compensatory skills. A qualitative study has provided support for the notion of compensatory skills in face-to-face CBT, where patients utilize the extensive self-therapeutic activities in CBT to manage their depression [11]. Self-therapeutic activity may involve the use of specific CBT techniques or personalized adaptations. Further, the patient might understand and cope with his/her depressive symptoms in light of new psychological knowledge. Similarly, self-therapeutic activity aimed at reducing negative cognitions may be one active ingredient in ICBT. Another line of psychotherapy research has shed light on contextual factors contributing to treatment outcome. The contextual model of psychotherapy [12] recognizes the contributions and interdependencies of other elements beyond the “bare-bones” treatment models and techniques (eg, mechanisms proposed by cognitive theory). These other factors include the actual patient and his/her expectations, factors outside the therapy situation (extra-therapeutic factors), and the working alliance between therapist and patient. The term working alliance refers to the partnership emerging between the therapist and patient in order to achieve the patient’s goal [13]. A robust relationship has been found between the quality of the working alliance—which depends on both therapist and patient factors—and outcome of treatment [14].

Although the effectiveness of computerized and Internet-based CBT is established, there is little agreement concerning the core content [15] and therapeutic process in self-help treatments [16]. Research on possible mechanisms of change has been emerging [9,16], and there is a need for studies aiming at furthering our understanding of active ingredients and processes at work in ICBT. Change in treatment specific factors for ICBT for depression, such as dysfunctional attitudes, worrying, and perceived control, has been found to mediate outcome [17]. Studies of the working alliance in Internet treatments report overall high ratings that are within the range of alliance ratings in face-to-face therapy [18]. Qualitative research on unguided ICBT has identified both CBT-related and patient aspects as influencing depression. Issues such as computer and Internet skills and the patient’s need for emotional support were reported as important influences during ICBT [6]. Qualitative research into guided ICBT yielded similar findings. It seems that the way patients work with CBT relates to the success of their outcome and their opinion about the therapy: an active, hands-on, self-reliant approach correlates with successful outcomes and favorable opinions. The opposite case is a passive style of working that does not put new knowledge into practice, skips parts of the course material, and is in need of more support [19]. It is not clear, however, what lies behind such differences in approach or whether the cause might be low expectations to the treatment, or because the patient feels a lack of helpfulness during the ICBT process. Purves and Dutton [16] explored patients’ experiences of the therapeutic process in an unguided computerized CBT program and identified four themes in interviews with patients. These included a meaningful relationship with the self-help material, using the self-help material to create structure to their psychological state, being stimulated by the self-help material creating engagement, and an increased sense of personal agency. In sum, the current literature implies that specific and common factors contribute to the outcome of ICBT. In order to improve effectiveness and acceptability of such treatment packages, further examination of these issues are warranted [9].

This study explores patients’ experiences of helpfulness in guided Internet-based cognitive behavioral therapy for depression. We intend to combine a pragmatic intention with a phenomenological-hermeneutical approach on patient experiences rather than on isolated patient factors. Such an approach, inspired by Husserl, focuses on human experience in everyday life, explores a natural attitude, and understands the whole of an experience [20]. Further, human beings have intentional relationships with their surroundings and things in their everyday lives, that is, relationships that are experienced as meaningful. In this case, we mean to elucidate how patients experience and give meaning to the phenomenon of ICBT in an everyday context. Such experience can be narrated and presented as text, which again calls for a hermeneutical interpretation [21]. To grasp the essential meaning of ICBT, this study aims to explore the experiences of patients in an ICBT intervention with therapist support. Knowledge about what patients experience as most helpful, and how patients understand and implement the principles of CBT, is essential if we want to improve the quality of ICBT programs and patient support. The current study aims to explore patients’ experiences with ICBT, focusing especially on those aspects of the therapy that they consider most helpful.

## Methods

### Design

This qualitative study was conducted in parallel with a randomized controlled trial (RCT; ACTRN12610000257066). The aim of the RCT was to test a treatment approach with ICBT that could be feasible in general practice. It compares Internet-based cognitive behavioral therapy interspersed with brief personal sessions with a therapist to a waiting list control sample. Participants in the RCT were recruited for this qualitative study when ending treatment. The first and second authors of this paper took part in the planning of the RCT and conducted the interviews. The first author worked as a therapist in the RCT but did not interview her own patients. Both the first and the second author conducting the interviews were blind to the outcome of the patients at the time of the interviews.

### Description of the Randomized Controlled Trial and Treatment

Patients included in the RCT after an initial assessment had a session with their therapist who introduced the self-help program. The introduction focused on giving brief information about the theoretical basis and the empirical support, as well as the content of the program and expected work load. Patients were asked to complete the five modules of MoodGYM in sequence, one per week. They were then followed up with weekly, face-to-face consultations with the therapist over a minimum of 7 weeks. A full course treatment included eight consultations. However, the treatment protocol was quite flexible and allowed for delays in the treatment, and it did not have a maximum limit for therapist sessions. In case of delays, the therapist contacted the patients to reschedule a new appointment.

The consultations each lasted approximately 15-30 minutes, similar to the time available in general practice. The guideline script comprised three compulsory subjects: (1) symptom monitoring, (2) discussion of the topic of the last module in MoodGYM, and (3) introducing the next module and discuss patient motivation. If time permitted, other issues that patients perceived as important to their depression were also discussed.

The self-help program used in the randomized controlled trial was MoodGYM. Its aim is to help patients prevent and cope with depression, based on principles of cognitive behavioral therapy [22,23]. It was developed at the Centre for Mental Health Research at Australian National University, and its effectiveness is empirically supported [24,25]. MoodGYM has five modules containing texts explaining the basic principles of CBT, a variety of self-tests and self-help exercises, and homework in which the patient is invited to analyze some personal experience in accordance with the principles of the program. Although some of the content in MoodGYM is generic CBT, there are also some specific sections devoted to parental relationships, relationship break-ups, problem solving, and even relaxation.

In the RCT sample, 72.6% were female, and age ranged from 18-63 with a mean of 36.1. The number of treatment sessions ranged from 1-12, with a median of 8 sessions; 40.1% did not complete the treatment program. The baseline depression scores, as measured by the Beck Depression Inventory (BDI-II) [26], had a mean of 21.7. A full description of the sample can be obtained in the forthcoming paper presenting the results of the RCT (personal communication by Høifødt, Ragnhild, March 2013).

### Recruitment to the Interviews and Procedure

Overall, the study was planned to provide complementary knowledge production on ICBT different from the kind of knowledge obtained in an RCT, hence the use of a qualitative approach. The recruitment of patients to the interviews was parallel to the randomized controlled trial, with patients receiving oral and written information about our qualitative study and an invitation to participate at their final consultation. Recruitment was continuous until the desired total of 14 interviews was reached. The recruitment procedure was strategic in the sense that we aimed to include men and women, younger and older, completers and noncompleters. The therapist would provide the interviewer with contact details for consenting patients. Patients could choose the location, either at their home or at the university in comfortable everyday like settings. All patients preferred to be interviewed at the university. The interviews lasted for approximately 60 minutes, were recorded using a digital voice recorder, transcribed verbatim by the second author or a clerical assistant, and then coded using NVIVO software. The initial coding procedure comprised a separate coding by the first, second, and last author of two interviews, and subsequently checked for consensus. During the entire process of analysis, the coding and the subsequent themes were discussed and reflected upon.

The researcher’s interview guide consisted of open questions inviting the patients to narrate different aspects of their ICBT experience: their motivation during progression, any changes they made in their everyday life, and any changes they perceived in their condition. This prompted answers concerning, eg, sociality, temporality, and spatiality of one’s lifeworld (the sum total of physical surroundings and everyday experiences that make up an individual’s world). As a whole, the interview was performed as an open dialogue interview. Table 1 presents a list of questions within each of the topic areas.

**Table 1:** Main questions in the interview guide.

Topic	Questions
Changes in everyday life	What was your life like before you got depressed? What was it like during your depression? How is it like after treatment? Can you describe any changes you have made during this time? Can you recall situations where you have acted differently as a result of the treatment? Do you think people close to you will have noticed any difference?
Motivation during treatment	What made you start treatment with MoodGYM? How did you progress through treatment? Which were important elements in treatment? Did you experience any difficulties?
The treatment	What did you think of the treatment? Was there anything you liked or disliked in particular? How did you like working with the computer? How did you experience the relationship with the therapist? In what way were you able to influence the progression through the treatment? Did you need to make any practical adjustment in your everyday life?

The qualitative study from the onset employed a phenomenological-hermeneutical methodology, which basically means that we sought to elicit the way participants related to the treatment. The approach is phenomenological in the sense that it understands human experience as founded in a basic relatedness to the world though merely living it in a naïve way, with a natural attitude. This experience, however, may be expressed in narratives, actions, or reflections, showing the intentionality of a human being, and may be described scientifically. This approach is inspired by Lindseth and Norberg [21] who again rest their methodology on both Husserl, Heidegger, and Ricoeur [21,27]. Semistructured interviews were conducted to elicit empirical information about patient experiences in treatment, aiming to set aside “the taken for granted” attitude of their lived experience.

### Patients

Fourteen patients were recruited from the RCT sample. The patients’ interviewed were 5 men and 9 women (64%), aged 22-61 years. The number of consultations ranged from 3-11. Three patients (21.4%) did not complete the treatment program. At pretreatment assessment, Beck depression inventory scores ranged from 10-28 (mean 18.27). At posttreatment assessment, 6 patients (42.8%) had not changed, 1 (7.1%) had improved, and 7 (50%) had recovered, based on criteria for clinically significant change [28].

### Analysis

Carrying out a phenomenological analysis requires the researcher to reflect carefully upon the taken for granted statements of one’s informants and to approach these with an attitude of “bracketing”, that is, to examine and question openly what is being expressed [20]. Also according to Lindseth & Norberg [21], a phenomenological-hermeneutical analysis cycles through different levels of understanding of the text material. First, the interview is read to achieve a superficial understanding of what the text is all about. Second, the text is analyzed in terms of its meaning units (semantics) with a reflective approach. The semantic units are then condensed to form themes and subthemes that disclose meaning in everyday words rather than merely portraying concepts. Also, the themes develop from the material rather than from the interview topics. The identified themes are, after the second step, compared with the initial cursory understanding for validation. Third, the themes are reviewed and reflected on as a whole, and an overall understanding may be reached through critical reflection based on theoretical literature. This procedure was adhered to, but it was necessary to compare the themes not only with the naïve understanding but also with the original interview transcript. And in the third step, comprehensive understanding was achieved in light of theories about change processes in psychotherapy. In summary, the analysis involved a dynamic process moving between abstracted themes and the interview transcripts for validation [27]. During the analytical steps, essential meaningful experiences with the treatment were identified and one of them was what counted as “helpful” for the patients. The themes revealing what helpfulness consisted of might emerge as pragmatic issues (which they were), but what was experienced as helpful was also interpreted as capturing exactly how the treatment became meaningful to the patients.

## Results

During analysis, several meaningful themes were identified from the interviews. The overall topic chosen for this paper is how patients perceive the treatment as being helpful. Another paper focuses on motivational aspects of the treatment (personal communication by Wilhelmsen, Maja, Jan. 2013).

Overall, we defined five themes reflecting perceived helpful dimensions. Two themes were related to being in treatment in general and were nonspecific to ICBT. These concerned (1) taking action to address one’s problems and (2) the value of talking to a professional. Two themes specifically addressed the patients’ experiences with the self-help program material: (3) acquiring relevant knowledge and (4) restructuring the new knowledge. A fifth theme concerned (5) the way that patients describe actual changes in perceptions and interactions with their environment that patients relate to the treatment they have undergone.

### Being in Treatment

#### Taking Action to Address One’s Problem

In most cases, patients had been considering the pros and cons of seeking help for a long time, even for years. One typical response to taking this difficult step was the sense of relief and satisfaction that patients felt when they took action to address their problem. Some patients had not gotten very far with the process and had not experienced any improvement, while others were highly active during treatment and making deliberate changes to their lives one step at a time. In both cases, patients talked about the mere act of doing something as important:

Working with MoodGYM, the best thing about it all was that I was doing something about it. You know, coming to these sessions every week, getting to talk, starting the next chapter. You know, the things I worked with did not suffice, but I felt good working with it. I felt sort of like I was getting out from...getting back to normal.

Male (26)

The analysis reflects the significance of moving from a state of passivity to one of activity. Patients recounted that they had felt a need to move forward that brought them to seek help. Taking action was in itself an achievement that engendered hope and brought a positive effect. The treatment might not be all they had hoped for, but patients still valued their own effort to try and improve their own situation. This theme is related to motivation in the treatment process, but it became evident in the interviews that this affected how the patients were feeling, as a meaningful event towards recovery.

Patients highlighted the easy access to the treatment as another important facilitator for taking action. The alternative for many people with depression would be referral to specialist health services, where long wait times leave patients passive. The alternative of a private clinic leaves patients worried about their financial liabilities. Our patients had low expectations about the abilities of general practitioners to deal with depression—GPs were assumed to have little time on their hands and little knowledge of mental illness. In general, our patients were pleased to be offered a course of qualified therapy universally available online at no cost.

#### The Value of Talking to a Professional

There was consensus among the interviewees that the involvement of a therapist was vital in the treatment and that talking to a professional is very important to them. The level of satisfaction with the amount of contact with the therapist varied a great deal. Some patients were quite satisfied, while others came to realize that what they really wanted was conventional face-to-face therapy, not guided ICBT. For all patients, a trusting relationship with a professional was a fundamental part of the treatment:

I thought it [the relationship with the therapist] was really good! She didn’t make me feel judged in any way. She was very accommodating. Almost as if she understood what I was talking about. She sometimes was ahead of me about things I was going to say, in a way. She understood very well what it was like.

Female (22)

Patients expressed a need to talk freely about their issues and a chance to reveal things about themselves without fear of judgment. Their need for appropriate verbal communication was significant. Communication with the therapist was described as different from everyday interaction with others, and patients thought it essential to the treatment. The chance for patients to share their experiences, innermost thoughts, and feelings was something they found important:

In the first session I got to talk about it all. So…the first session was very important…the first and the second session, that was when I got to talk about the things that troubled me. So I believe…I think it was very important to be able to do that.

Male (26)

Furthermore, the reciprocity of the relationship was emphasized: not in the sense that the therapist and the patient are equal, rather, the role of the therapist as a professional was appreciated. The importance of a dialogue was stressed, where the patient could ask questions, discuss issues with the therapist, receive trustworthy feedback, and be supported and acknowledged:

But, I have sort of looked for…what shall I say, a professional or an adult who has in some way supported me in my thoughts about all the things I do. Those things I do because I feel guilty, have to, but in reality don’t need to. I would like a verbal confirmation that I’m doing enough!

Female (56)

These aspects of the professional relationship, that is, having someone you can trust, a chance to freely express yourself, and receiving individual affirmation, were for some just as important as working with direct symptom relief or negative thought patterns. It seems that by engaging in this dialogue, these patients were reassured by the affirmation and support they received.

### Internet-Based Cognitive Behavioral Therapy

One vital aspect of MoodGYM depression therapy is that patients are actively engaged; they receive homework aimed at challenging their repetitive automatic negative thoughts and cognitive distortions. The latter two themes: (3) acquiring relevant knowledge and (4) restructuring the new knowledge, describe ways in which the content of MoodGYM was experienced by patients. The extent to which patients could relate to MoodGYM varied greatly, so these responses provided insight into relevant dimensions of interacting with the program.

#### Acquiring Relevant Knowledge

Patients commonly describe features of the self-help material as being experienced as particularly relevant, issues presented in the program that “have to do with me”, or in contrast, issues that were irrelevant. Typically, patients were more satisfied with the standard content of CBT (eg, presenting the principles, cognitive distortions) rather than the content aimed at specific problems (eg, relationship break-ups, parental relationships). Patients could accept some parts of the content being of little relevance to them, as long as they could find other parts that they could learn from:

The first thing I came to the session and said, “this, this is huge”. It [a presentation of the basic ABC-model of CBT] was the drawing we saw early on, with situations, cognitions and feelings. I had never seen that drawing before…and if I were to draw it, feelings would never be in such a drawing. Because I’ve ignored feelings…So that was, well, a first…a sort of awakening.

Female (39)

The fact that a patient could recognize something in the program content and feel its relevance was a way to feel support and recognition in a situation that otherwise was unfamiliar or hard to accept. The process of seeing relevance in the material is intertwined with the process of learning and acquiring new self-knowledge. Conversely, patients who found no relevance in the material were unlikely to learn anything. So for some respondents, the content raised awareness, reactivated once-familiar knowledge, or provided new insights; whereas for others, the feeling was that the content was not meaningful to them:

Well, so little by little, when I could only identify with the character that was not depressed, then it like became more and more…it was almost as if I felt myself getting annoyed by those modules. And I decided that this here stuff doesn’t give me anything.

Female (28)

Not all patients felt able to relate to the principles of CBT presented in MoodGYM. This was generally due to a mismatch of the program aims and what patients perceived as their most pressing problem. All our patients showed symptoms of depression, but not all of them felt depressive thoughts and ideas to be their principal problem. Some were quite clear in their mind that ICBT would not provide the answer to their difficulties, perhaps to the extent that they were unwilling or unable to relate to the MoodGYM content.

#### Restructuring the New Knowledge

Patients’ accounts of their experiences with the program content show how they reflected on the material, adapting or processing it to suit their own perceived needs. Patients who found parts of the self-help program relevant did not necessarily accept it all uncritically, rather, they described an active process of interpreting the material. This also meant reflecting on past events, thoughts, and feelings in light of their newfound understanding:

I felt it [working with the modules] took a long time because I was sitting reading and trying to interact…interact with what I read…It was not that I struggled with the homework or with understanding what it said, but I chose to spend time on it.

Female (26)

**Figure 1:**
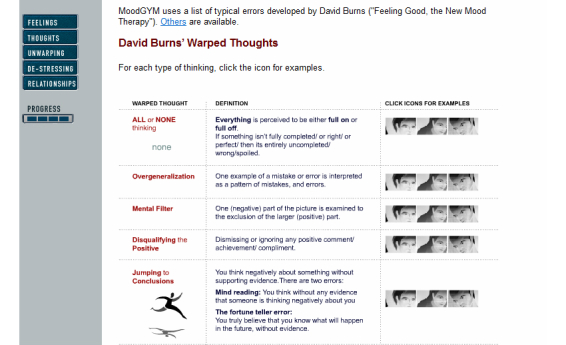
Screenshot from module 2 in MoodGYM.

It was evident that the proposed approaches and techniques were not universally suitable, and some patients went to considerable lengths to restructure the content to suit their perceived needs. A table in module 2 gives an overview of distorted cognitions (Figure 1).

There were an awful lot of categories. How could I make it useful? On a daily basis, I had to merge some into larger groups and then work out, like, “Alright, now I’m making this type of mistake”. It had to be restructured a bit, because I couldn’t be bothered to sit and cram all of them. And I didn’t need to either.

Male (33)

The patients talked about how the program material made them more aware of their own negative thinking and that this awareness opened up for further reflections about the validity of those thoughts, and how such thoughts are incorporated in a negative cycle that also includes feelings and actions. However, not all patients were able to make adaptations of the material to fit their own needs, despite recognizing the relevance of the material. For some, bridging the gap between theoretical concepts of negative thinking and making the ideas their own, was far from easy. Sessions with the therapist helped the bridging process by enhancing patients’ understanding and ideas about program content: “Indeed the conversations helped make the content of the Internet form more elastic”. [Female (39)]

#### Actual Changes in Perceptions and Interactions

Patients described changes in both thought and behavior as essential. Some changes were clearly related to what they learned from the self-help program, eg, testing the truth value of their negative thinking or using specific techniques from the program, whereas other changes had been discussed with the therapist in the sessions and were not directly related to the self-help program. Changes in thought patterns, where patients started to question the content and validity of their depressive ideas, were a specific result of the self-help techniques:

And also in relation to the business of structuring and categorizing thoughts and mindsets, and doing a reality check on what you’re thinking, related to what has happened or what you’re feeling—that has been very helpful to me. What does this really mean? Where does this come from? This has been very useful to me.

Female (39)

**Figure 2:**
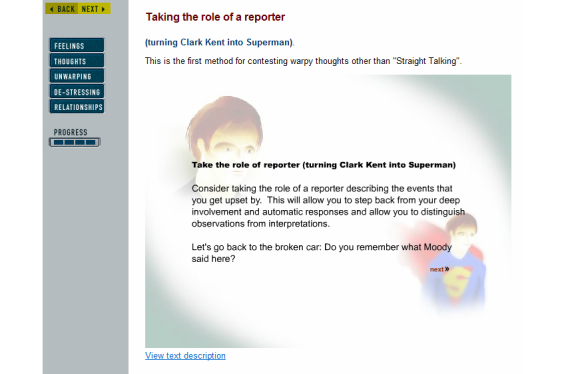
Screenshot from module 3 in MoodGYM.

The patients were describing how they came to realize that thoughts and ideas about an event are not accurate representations of reality. Moreover, they might be biased and need careful scrutiny. Questioning the validity of thoughts and ideas made room to explore other possible interpretations, and this process helped give patients more flexibility in their thinking. It showed, for example, how a bias in their mindset might be making their depression worse. Behavioral changes could follow such cognitive changes, including how patients related to events and how they discussed things with others. A technique in module 3 distinguishes observations from interpretations (Figure 2). The following quote illustrates how an exercise from MoodGYM (“The Reporter”) is incorporated by one patient:

There were some things I remember, and that was when I got that far in the program, then they talked about a reporter. I’ve given that much thought, and that’s something I’ll take with me. Actually, I am an emotional person, and sometimes I get annoyed with myself…And then there was this here reporter…her name is Vold. She [a Norwegian television reporter] reports from the Middle East and Palestine, and I think she’s good. And I imagined her in front of me…she’s in the midst of fighting, or they are shooting, or they are fighting all around her, or at least in the background. And she’s standing there trying to report accurately on what has happened. I think it’s admirable. So I’ve had her with me in quite a few situations.

Female (61)

Other behavioral changes served to break a negative cycle of self-recrimination, inactivity, and withdrawal. These changes could produce positive secondary benefits for the patients, including getting closer to the people around them:

I got to know her [his partner’s] kids better, for instance. That’s very positive. [Interviewer: “How did that happen?”] Well, I tried to make the best of it and play with them, not going into a room and hiding away all by myself.

Male (26)

Some changes described by the patients revealed that their self-perception had tended to move in a positive direction, towards self-acceptance. This was evident from statements that revealed a more self-accepting and less self-critical attitude towards themselves:

If you have done something that you’re not completely satisfied with, you should not think that you’re a terrible person. That you can actually get a firm grip on it and work with it.

Female (41)

Some of the changes patients made in their daily lives sprang out of the sessions with the therapist, but were not directly related to any of the program material. Examples might be practical solutions to problems discussed in the sessions, or specific strategies to break a negative cycle, designed specifically for the patient’s own situation: “Yes, we had to find practical solutions, because it’s not always that positive thinking can silence the negative.” [Female (22)]

This is an illustration that patients could also experience other difficulties in their lives that needed attention. Examples are practical problems that needed to be tackled, in addition to the cognitive behavioral therapy provided in the self-help program.

## Discussion

### Principal Findings

The previous section has looked at what patients experienced as helpful with regards to treatment. These comprised basic dimensions as the very act of seeking help and being in a therapeutic relationship with a professional. Patients described their experiences of the specific CBT delivered by MoodGYM as a source of relevant knowledge that they adapted to their own situation and implemented in their everyday lives. Overall, the phenomenological-hermeneutical analysis gave knowledge of several experiences with ICBT concerning helpfulness but also provided a whole impression of how these experiences were related and understood. The patient identifies with and learns from MoodGYM material that stimulates them to engage in self-therapeutic activity. This process is supported through the consultations with the therapist. In addition, the relationship with the therapist had a function beyond supporting MoodGYM use, in providing an arena for sharing thoughts and feelings and receiving feedback and advice.

Regarding help-seeking, patients described that making a commitment to therapy was a great help suggesting that dimensions within the patient, not directly related to treatment content, have an influence on outcomes. Taking action to improve one’s situation is a way of regaining control, referred to by previous authors as empowerment [16] or being compelled to take action [29,30]. Taking action would include making a commitment to the ICBT treatment and accepting its methodology, which again can be interpreted as an expression of the treatment relationship or the working alliance. The working alliance is shaped within the context of the therapy and represents the bond between therapist and patient, as well as a shared understanding of the work and goals of the treatment [13,31,32]. The patients were given an explanation of ICBT, and most accepted self-help therapy as a way forward with themselves being the primary agent of change. Yet there were also cases where the commitment was poor, and the patient did not accept the basic premises of ICBT or deemed the content of MoodGYM inappropriate to their needs. This resulted in poor motivation and little active engagement in the treatment. The success of the working alliance is strongly related to the success of the therapy outcome [33,34], which indicates that establishing a common understanding of the aims of the treatment is a vital key to recovery. There are findings pointing to the ability of patients to develop a meaningful relationship within a fully computerized treatment [16]. However, research findings concerning the working alliance in ICBT are inconclusive [18], and further research, particularly in relation to the type of support (face-to-face, telephone, or electronic messages) and treatment outcome is warranted.

The presence of a therapist was important in several ways. It means a trusting relationship can be developed, and patients have a channel for self-disclosure and supportive response. The need for disclosing thoughts and feelings, interaction, and feedback has been highlighted in previous studies [6,29,35], pointing to the role of the therapist in the therapeutic relationship as warm, empathic, affirming, and engaging [36]. In a previous study, patients reported difficulties in translating computerized CBT content to their own social situation [6]. In the current study, sessions with the therapist added to the self-help program by opening up for discussion of the program content, assisting the patients in their understanding of the content. As such, the role of the therapist influenced the specific CBT dimensions. As in previous studies, some patients deemed the sessions confined and desired more conversation with a therapist to gain a deeper understanding of their problems [19,29]. Another benefit was that the therapist could step in when problems were beyond the scope of the program or tender helpful practical advice tailored to the individual. Clinicians consider a lack of these possibilities as potential drawbacks of ICBT [37]. Thus, patients and therapists value the flexibility and possibility to individually adapt the intervention in a way only human support can provide.

Acquiring new knowledge was a significant benefit from involvement in the MoodGYM program. Patients described themselves being able to relate to cases presented in the program. Patients actively sought out the parts of the program helpful to them and found ways to utilize this new knowledge. The findings show that these patients were not simply passive recipients of insights gleaned from MoodGYM, rather they were active seekers of relevant information, evaluated its validity, and adapted their new knowledge to their own personal situation. These findings are consistent with the perspective of patient involvement in psychotherapy [38] and with previous research where the self-help material stimulates new learning, making patients able to create more structure and order, and break problems down to smaller entities [6,16,19,30]. Within this perspective, the patient is an active agent, entering therapy with some ideas of what they need, selecting from therapy what they consider useful, making independent assessments of results, and integrating therapy experiences into everyday life.

Patients talked about self-therapeutic activities in terms of implementing their own adaptations of MoodGYM content and discussions with the therapist. Commonly, patients reported general insights and increased awareness of negative automatic thoughts and cognitive distortions in real life, similar to previous observations in ICBT [19] and face-to-face CBT [11]. For some patients, these insights enabled them to challenge the validity of such unruly thoughts. Changes in behavior and communication could bring secondary benefits, further strengthening the positive processes in play. Some changes were subtler, not being specifically bound to any given situation but seemed to represent a shift in self-esteem across all situations, with the patient becoming more self-accepting and less judgmental.

In light of the findings from the current qualitative study, guided ICBT with MoodGYM can be viewed as a dynamic process, in which the patient and the therapist work together within the self-help framework offered by the online treatment program.

### Implications of the Study

The results of this study highlight how ICBT can be a useful treatment for depression by providing insight into everyday life experiences with this mode of treatment. This has brought forward both patient-near experiences and pragmatic solutions to identified problems. The results specifically illustrate the dynamic interplay between the patient’s lifeworld, the therapist, and the ICBT treatment program. This is important to keep in mind for the future development and implementation of guided self-help.

The collaborative nature of guided ICBT is evident from the results of this study. The patient plays an active role in the therapeutic process, and this point should be explicitly stated before and during the course of the treatment. Needless to say, individual patients have different capacities to get involved, some needing more support and encouragement than others.

The ease with which patients could adapt standard principles to suit their own situations varied a great deal. This realization should help inform continuing development of online self-help programs. In general, generic CBT modules were experienced as more relevant than modules targeting specific issues (eg, problem solving, relaxation), supporting previous findings [39]. Principles and descriptions in the program should be generic to the extent they are recognizable to the patient, yet designed to work with patients with a specific diagnosis [40], but adaptable to an everyday context. This precise diagnosis or description of the patient’s problem needs to be prepared beforehand, allowing the patient to be matched to the most suitable program package [7].

Whether therapist support is provided through email, telephone calls, or face-to-face, it can be assumed to contribute to the outcome of the therapy directly and indirectly. Therapist support should offer the opportunity for a level of self-disclosure, in a nonjudgmental setting where patients can expect relevant, supportive feedback. The therapist also serves an important function to help patients understand the principles of CBT and help patients make the transformation from principle to everyday practice.

The role of the therapist was also vital when patients needed individual guidance or advice about how to deal with everyday challenges that might otherwise get in the way of treatment progress. Practical advice from a therapist, or an inspirational session with a sympathetic adult, could provide the motivation to embark on manageable, significant changes.

The findings of the present study point to the dimensions experienced as important by the patients interviewed, and the meaning of this mode of treatment in terms of moving from depression towards recovery. These findings may serve as a hint on how to continue to improve the practice of ICBT. It remains to explore what conditions in ICBT are sufficient and necessary to aid patients, and what distinguishes processes resulting in change from those ending in no change or deterioration.

### Limitations

The sample of patients in this study is small, consisting of individuals who volunteered to join a randomized controlled trial and attend the interviews. Possibly they were highly motivated to participate in research, and they are in some way different from other potential candidates who chose not to be interviewed. Furthermore, recruiting noncompleters turned out to be difficult, and thus the completers are overrepresented in the sample. This is a limitation of the study. It is likely that completers and noncompleters have divergent views regarding the helpfulness of the treatment, and we recommend further research into the treatment experiences of noncompleters of ICBT. However, there were differences within the sample regarding outcome, and accordingly, the results also reflect the views of patients who did not improve.

The study examined patients’ experiences with an online self-help program, incorporating occasional, brief face-to-face sessions with a therapist. By contrast, Internet-based therapy typically gives guidance through electronic messages or telephone calls. It is possible that the treatment examined in the current study has a greater resemblance to conventional psychotherapy than to Web-based self-help. Further investigation could identify other possible themes for discussion in other forms of Web-based self-help therapies.

The interviews were conducted shortly after the end of treatment, therefore it is not possible to evaluate to what extent potentially helpful elements impacted on patients’ lives. It may be that patients were still “fired up” by the therapy at the time of the interview, but this does not necessarily mean they experienced any long-term improvements. Some findings suggest that patients can continue to engage in self-therapeutic CBT for as much as 3 months after ending treatment [11], but further research is needed to shed light on patient agency in CBT.

### Conclusion

Elements in ICBT that are perceived as helpful represent the essence of the patients’ experience with ICBT. This can be viewed from the perspective of the contextual model of psychotherapy, which highlights the dynamic and collaborative nature of Internet-based self-help. The findings of the current study pointed to MoodGYM as a source of relevant knowledge, providing a structured approach to working with depression. The role of the patient as the primary agent of change is highlighted, through his/her engagement in treatment, seeking knowledge, and employing it to the personal context. During the intervening guidance sessions, the therapist played a useful role by facilitating the understanding and explaining the relevance of the generic MoodGYM content, providing professional feedback and interpersonal support, as well as giving practical advice.

## Abbreviations

BDI: Beck Depression Inventory
CBT: cognitive behavioral therapy
ICBT: Internet-based cognitive behavioral therapy
RCT: randomized controlled trial
